# Gd-EOB-DTPA-Enhanced Magnetic Resonance Imaging for Assessing Liver Function in Primary Biliary Cholangitis

**DOI:** 10.2174/0115734056379090250611073434

**Published:** 2025-06-23

**Authors:** Zhengjun Li, Fan Zhang, Weiting Lu, Chao Lu, Zheng Yuan, Zhongqiu Wang

**Affiliations:** 1 Department of Radiology, Affiliated Hospital of Nanjing University of Chinese Medicine, Nanjing, Jiangsu, China; 2 Department of Infectious Diseases, Affiliated Hospital with Nanjing University of Chinese Medicine, Nanjing, Jiangsu, China

**Keywords:** Magnetic resonance imaging, Gd-EOB-DTPA, SWE, Primary biliary cholangitis, Liver function, Portal venous phase

## Abstract

**Introduction:**

This study aimed to detect the performance of gadolinium ethoxybenzyl diethylenetriamine pentaacetic acid (Gd-EOB-DTPA)-enhanced magnetic resonance imaging (MRI) for assessing primary biliary cholangitis (PBC).

**Methods:**

Seventy-five patients with PBC were included in this prospective study. Shear wave elastography (SWE) and Gd-EOB-DTPA-enhanced MRI were conducted, and then the signal intensity ratio (SIR) and contrast enhancement index (CEI) in different phases, including portal venous phase (PVP), equilibrium phase (EP), and hepatobiliary phase (HBP), were calculated. Afterward, the results were compared with Child-Pugh grading and non-invasive liver fibrosis models using the Kruskal-Wallis H test or Chi-squared test. The area under the curve (AUC) was applied to evaluate the diagnostic performance of SIR_HBP_, CEI_HBP_, and SWE across different Child-Pugh grades.

**Results:**

SWE (*p*<0.001), SIR _HBP_ (*p*<0.001), CEI_HBP_ (*p*<0.001), APRI (*p*=0.002), and FIB-4(*p*<0.001) showed significant differences in different Child-Pugh grades. Statistically significant differences were found in SIR_HBP_ (*p*=0.005), CEI_HBP_ (*p*=0.010), and FIB-4 (*p*=0.001) of different SWE levels. For the diagnosis of Child-Pugh C, the AUC of SWE, SIR_HBP,_ and CEI_HBP_ were 0.889, 0.778, and 0.761, respectively. Correspondingly, the sensitivity was 75.0%, 64.4%, and 54.2%, and the specificity was 94.9%, 100%, and 100%, respectively. For the diagnosis of Child-Pugh B+C, the AUC of SWE, SIR_HBP,_ and CEI_HBP_ were 0.919, 0.809, and 0.814, respectively.

**Discussion:**

Our study confirmed that Gd-EOB-DTPA-enhanced MRI is an effective and objective method for assessing liver function in patients with PBC.

**Conclusion:**

SIR_HBP_ and CEI_HBP_ could be regarded as a novel imaging biomarker to evaluate liver function. Gd-EOB-DTPA-enhanced MRI and SWE outperformed serum-based models in sensitivity and specificity, strengthening the value of imaging in clinical decision-making.

## 1 INTRODUCTION

Primary biliary cholangitis (PBC) is the most common autoimmune liver disease, characterized by chronic inflammation of small bile ducts [1]. It may result in fibrosis, cirrhosis, and eventually liver failure or liver cancer. The global prevalence of PBC is approximately 14.60 per 100000 population, while the incidence in the Asian-Pacific region has been slowly increasing [2, 3]. Untreated PBC progresses at an average rate of one histological stage every 1.5-2 years. Treated individuals have a fivefold decreased rate of progression to cirrhosis once diagnosed at an early histologic stage.

Histological staging and grading systems for PBC, such as Scheuer’s or Ludwig’s classifications, have been widely used [4]. However, as invasive examinations, they carry certain risks and poor reproducibility. EASL Clinical Practice Guidelines on non-invasive tests for assessing liver disease severity and prognosis stated that serum biomarkers are not recommended for fibrosis staging in patients with PBC [5]. Transient elastography is proposed as a standard for identifying severe fibrosis [6]. Moreover, several novel deep learning networks would provide insight into improving medical imaging quality, which is crucial for magnetic resonance imaging (MRI) assessment of liver conditions and applicable to liver imaging techniques [7, 8]. In order to overcome these limitations, there is substantial clinical value in exploring non-invasive and repeatable indicators for assessing liver function in PBC patients.

Therefore, in the current study, real-time shear wave elastography (SWE) and gadolinium ethoxybenzyl diethylenetriamine pentaacetic acid (Gd-EOB-DTPA)-enhanced magnetic resonance imaging (MRI) were applied in assessing PBC, and their results were compared with different Child-Pugh grades. Furthermore, the distribution characteristics of non-invasive fibrosis models, including aspartate transaminase to platelet ratio index (APRI), fibrosis index based on the 4 factors (FIB-4), gamma-glutamyl transferase to platelet ratio (GPR), were examined in PBC patients, in order to provide further evidence in characterizing the severity of PBC patients using SWE, Gd-EOB-DTPA-enhanced MRI, and these non-invasive fibrosis models.

## 2 MATERIALS AND METHODS

### 2.1 Participants

Patients with PBC at Jiangsu Province Hospital of Chinese Medicine from January 2021 to September 2023 were included in this prospective observational study. All procedures involving human subjects complied with the declaration of Helsinki. Each participant provided written informed consent before participating in this study, which was approved by the Ethics Committee of the Affiliated Hospital of Nanjing University of Chinese Medicine (approval number: 2021NL-101-02).

Based on the guidelines of the American Association for the Study of Liver Diseases (AASLD) [9], PBC patients were diagnosed according to two or more of the following criteria: (1) Serological cholestasis changes with elevated levels of alkaline phosphatase (ALP) or gamma-glutamyltransferase (GGT), without interpretable causes; (2) Presence of positive anti-mitochondrial antibodies (AMA); and (3) Liver biopsy showing non-suppurative cholangitis as well as destruction of the medium and small bile ducts. All enrolled patients received oral ursodeoxycholic acid (UDCA) therapy. Exclusion criteria were as follows: (1) Large liver mass lesions; (2) Pregnant or lactating women; (3) Under 18 years old; (4) Contraindications for MRI; (5) Allergy to Gd-EOB-DTPA; (6) Concurrent factors causing chronic liver diseases; and (7) The presence of other systemic illnesses. The flowchart of the study population is shown in Fig. (**1**).

### 2.2 Laboratory Tests

A complete blood count test was conducted to assess platelets (PLT) as well as prothrombin time and measured various enzymes and markers, including aspartate transaminase (AST), alanine transaminase (ALT), alkaline phosphatase (ALP), and gamma-glutamyl transferase (GGT). Based on these biological parameters, Child-Pugh scores were calculated as follows: Child-Pugh A: 5 to 6 points, Child-Pugh B: 7 to 9 points, and Child-Pugh C: 10 to 15 points. The non-invasive fibrosis models were calculated as follows:

APRI=[AST/upper limit of normal× 100]/PLT(×10^9^/L);

FIB-4=age(years)×AST(U/L)/[PLT(×10^9^/L)×ALT(U/L)^1/2^];

GPR=[GGT(U/L)/upper limit of normal×100]/PLT (×10^9^/L).

The upper limit of normal AST is 32 U/L.

The upper limit of normal GGT is 40 U/L.

### 2.3 Imaging Methods

MRI data were acquired with a phased-array coil using a GE Architect 3.0T scanner (GE Healthcare, USA) from the Department of Radiology. For MRI enhancement, 25 μmol/kg Gd–EOB–DTPA (Bayer, Germany) was injected at a speed of nearly 1 mL/second through peripheral veins. Dynamic 3D-T1 fast-field-echo sequence was carried out before [precontrast phase] as well as after 60 seconds [portal venous phase (PVP)], 300 seconds [equilibrium phase (EP)], and 20 minutes [hepatobiliary phase(HBP)] following the contrast-agent injection, with relevant parameter, such as TR/TE: 3.2/1ms; slice thickness: 4mm; matrix: 320 × 242; NEX: 1; flip angle: 15°; and FOV: 38 cm.

### 2.4 SWE Methods

The SWE was performed with Supersonic Axplore (The SC6-1 type probe) under calm breathing. In a right intercostal transducer position, large vessels and ascites were avoided. A circular region of interest (ROI) with a 1 cm diameter was then placed in the elastogram at least 1 cm below the liver capsule at a depth of 3 to 5 cm. The median of the five successful SWE (kPa) values was selected as the final result.

### 2.5 Imaging Analysis

Two experienced radiologists with more than 15 years of experience in MRI reviewed Gd-EOB-DTPA-enhanced MRI images. Any different opinions between two radiologists would be resolved by a third radiologist who was also blinded to clinical information.

The signal intensity (SI) values of the liver were measured on PVP, EP, and HBP T1-weighted images using ROI. After excluding liver lesions, visible blood vessels, or imaging artifacts, three ROIs in the liver were outlined in each phase (two in the right lobe and one in the left lobe). The two ROIs in paravertebral muscle (PM) on pre-contrast images were outlined with an area of at least 200 mm^2^, and the measurements were averaged. The signal intensity ratio (SIR) and contrast enhancement index (CEI) in different phases were calculated as: (1) SIR_PVP_=SI_PVP_/SI_PM_, (2) SIR_EP_=SI_EP_/SI_PM_, (3) SIR_HBP_=SI_HBP_/SI_PM_, (4) CEI_PVP_=(SI_PVP_-SI_pre_)/SI_PVP_, (5) CEI_EP_=(SI_EP_-SI_pre_)/SI_pre_, and (6) CEI_HBP_=(SI_HBP_-SI_pre_)/SI_pre_. Different phases are depicted in Fig. (**2A**-**E**).

### 2.6 Statistical Analysis

Statistical analysis was performed using GraphPad Prism 8.3 and SPSS Statistics 25 software. The median (P_25_, P_75_) was used to summarize continuous data with a non-normal distribution. Numbers (percentages) were used to show categorical data. Kruskal-Wallis H test was conducted for non-normally distributed continuous variables, and the Chi-squared test was carried out to compare categorical variables among groups. Pearson correlation analysis was performed for SIR_HBP_, CEI_HBP_, and SWE. Finally, the area under the receiver operating characteristic (ROC) curve was used to assess diagnostic performance and compare the ROC of SIR_HBP_, CEI_HBP,_ and SWE across different Child-Pugh grades. Two-tailed *p*<0.05 was considered statistically significant.

## 3 RESULTS

### 3.1 Clinical Characteristics

A total of 75 PBC patients were eligible for final analysis. The majority of them were females (n=62, 82.7%). The clinical characteristics of PBC patients are presented in Table **1**.

### 3.2 Comparison of Gd-EOB-DTPA-enhanced MRI, SWE, and Non-invasive Fibrosis Models Across Different Child-Pugh Grades

Patients with advanced Child-Pugh grade showed significantly decreased SIR and CEI levels and elevated SWE, APRI, and FIB-4 levels compared to those with early Child-Pugh grade (all *p* < 0.01, Table **2**).

### 3.3 Comparison of Gd-EOB-DTPA-enhanced MRI, Non-invasive Fibrosis Models, and Child-Pugh Grades Across Different SWE Grades

**Fig. (3):**
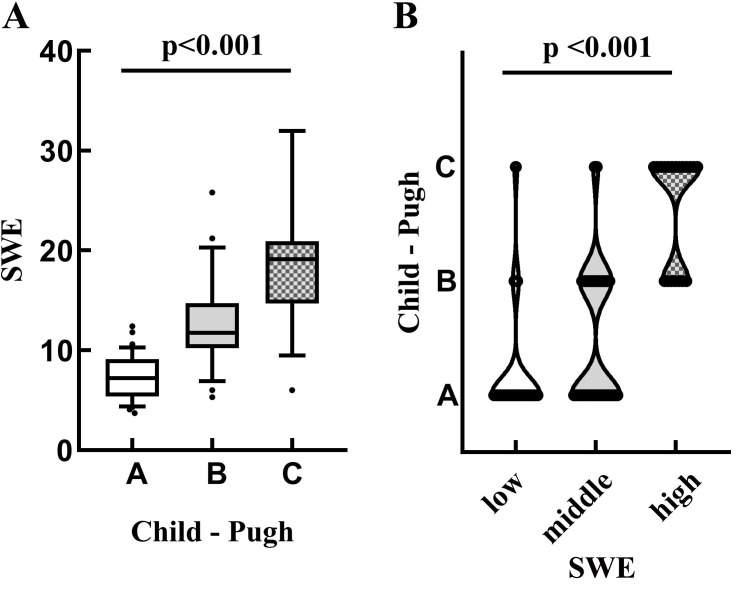
(**A**) Comparison of SWE values in PBC patients with different Child-Pugh grades. (**B**) Distribution of Child-Pugh grade in patients with different SWE levels.

All the PBC patients were divided into three groups according to the SWE scores: SWE≤7.0, 7.0<SWE≤14, and SWE>14. Patients with high SWE scores showed decreased SIR and CEI levels. The FIB-4 of the highest SWE grade was significantly higher than the other two SWE grades (*p* < 0.01, Table **3**). As SWE values increased from low to high, the proportion of patients classified as Child-Pugh C stage also increased (*p* < 0.01) (Fig. **3A**-**B**).

### 3.4 Comparison of SWE, Non-invasive Fibrosis Models, and Child-Pugh Grades Across Different CEI_HBP_ Grades

**Fig. (4):**
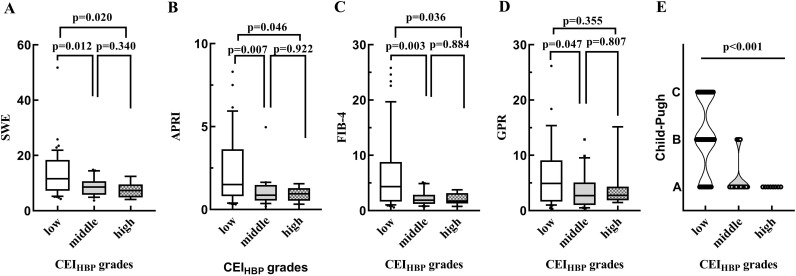
Distribution of SWE (**A**), APRI (**B**), FIB-4 (**C**), GPR (**D**), and Child-Pugh (**E**) according to CEI_HBP_ grades.

All the PBC patients were divided into three groups according to the CEI_HBP_ scores: low: CEI_HBP_≤1, middle: CEI_HBP_: 1< CEI_HBP_≤2, and high: CEI_HBP_>2. The SWE, APRI, and FBI-4 scores were lower in the low CEI_HBP_ group than in both middle and high CEI_HBP_ groups (*p* < 0.05). As CEI_HBP_ values increased from low to high, the proportion of patients classified as Child-Pugh C stage also decreased (*p* < 0.001) (Fig. **4A**-**E**). No statistically significant differences were identified across different CEI_HBP_ grades concerning age and sex (*p* > 0.05).

### 3.5 Correlations between SWE, SIR_HBP_ and CEI_HBP_

**Fig. (5):**
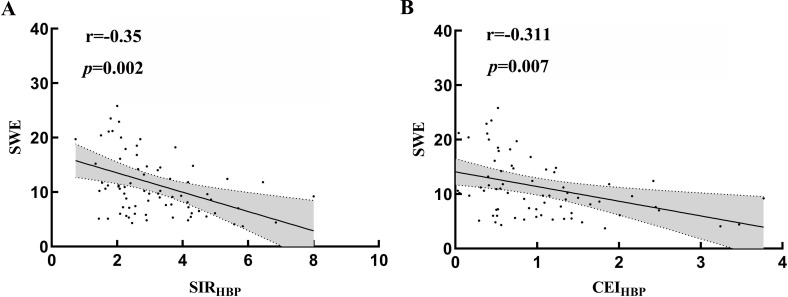
Correlations among SWE, SIR_HBP_, and CEI_HBP_. SWE was negatively correlated with SIR_HBP_ (r =-0.351, *p* =0.002) (A) and CEI_HBP_ (r =-0.311, *p* = 0.007) (B).

Pearson’s correlation analysis was carried out to analyze the correlations between SWE, SIR_HBP_, and CEI_HBP_. SWE was negatively correlated with SIR_HBP_ (r =-0.351, *p* =0.002) and CEI_HBP_ (r =-0.311, *p* = 0.007) (Fig. **5A**,**B**).

### 3.6 Diagnostic Value of SWE, SIR_HBP_, CEI_HBP,_ and Non-invasive Fibrosis Models Across Different Child-Pugh Grades

**Fig. (6):**
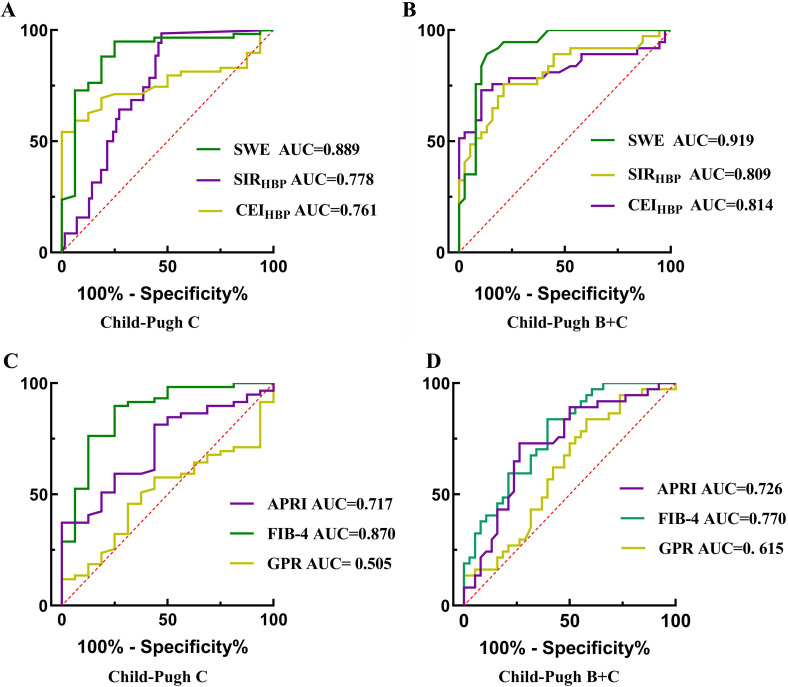
The ROC analysis of SWE, SIR_HBP,_ and CEI_HBP_ for the identification of Child-Pugh C (**A**) and Child-Pugh B+C (**B**). The ROC analysis of APRI, FIB-4, and GPR for the identification of Child-Pugh C (**C**) and Child-Pugh B+C (**D**).

ROC curve was used to evaluate the diagnostic value of SWE, SIR_HBP,_ CEI_HBP,_ and non-invasive fibrosis models across different Child-Pugh grades in patients with PBC (Fig. **6A**-**D**). For diagnostic accuracy of Child-Pugh C, the AUCs of SWE, SIR_HBP,_ and CEI_HBP_ were 0.889, 0.778, and 0.761, respectively. Correspondingly, their sensitivity was 75.0%, 64.4%, and 54.2%, and the specificity was 94.9%, 100%, and 100%, respectively. The AUCs for differentiating patients with Child-Pugh A from those with Child-Pugh B and C of SWE, SIR_HBP_, and CEI_HBP_ were 0.919, 0.809, and 0.814, respectively. Correspondingly, the sensitivity was 86.8%, 75.7%, and 73.0%, and the specificity was 89.2%, 78.9%, and 89.5%, respectively.

For diagnostic accuracy of Child-Pugh C, the AUCs of APRI, FIB-4, and GPR were 0.717, 0.870, and 0.505, respectively. Correspondingly, the sensitivity was 56.3%, 75.0%, and 62.5%, and the specificity was 81.4%, 89.8%, and 52.5%, respectively. The AUCs for differentiating patients with Child-Pugh A from those with B and C of APRI, FIB-4, and GPR were 0.726, 0.770, and 0.612, respectively. Correspondingly, the sensitivity was 73.7%, 60.5%, and 42.1%, and the specificity was 73.0%, 83.8%, and 83.8%, respectively.

## 4 DISCUSSION

Gd–EOB–DTPA is a hepatocyte-specific contrast agent used in magnetic resonance imaging (MRI) that combines perfusion information with hepatocyte-selective properties [9], which has become a pivotal diagnostic modality for liver cancer. After injection of Gd-EOB-DTPA, 50% of it is absorbed by normal liver cells after 10–20 min through the organic anion transporting polypeptide 1B3 (OATP1B3) located on the liver cell membrane of the hepatic sinusoids [10, 11]. It is then excreted into the biliary tract through multidrug resistance-associated protein 2 (MRP2) on the biliary tract of the hepatic membrane, which is called the hepatobiliary phase. Reduction of normal liver cells or dysfunction of the OATP1-MRP2 pathway can both lead to decreased absorption of Gd-EOB-DTPA, resulting in variations in hepatobiliary enhancement [12, 13], which may be related to the Child-Pugh classification.

The current results showed that patients with higher Child-Pugh levels had decreased SIR_HBP_ and CEI_HBP_ levels, indicating that Gd-EOB-DTPA enhancement is influenced by the Child-Pugh classification. This suggests that, despite differing etiologies of liver damage, the intensity of Gd-EOB-DTPA during the hepatobiliary phase similarly reflects liver function. Compared with the traditional liver reserve function test ICG-R15, the pooled correlation coefficient (r) between the reduction rate of T1 relaxation time in HBP and ICG-R15 was -0.47, and the liver-to-muscle ratio of HBP was negatively correlated with ICG-R15 [14, 15].

Our study reported that for the diagnostic accuracy of Child-Pugh B and C, the AUCs and specificity of CEI_HBP_ (0.814 and 89.5%, respectively) were higher than those of SIR_HBP_ (0.809 and 78.9%, respectively). The specificity of SIR_HBP_ and CEI_HBP_ for diagnosing Child-Pugh C was 100%, although their sensitivity was not as high. A prior study demonstrated that the AUCs of Gd-EOB-DTPA-enhanced MRI for the detection of liver fibrosis at stages F1 or greater, F2 or greater, F3 or greater, or F4 were 0.8669, 0.8399, 0.8481, and 0.8858, respectively [16]. T1 relaxation time decreased with the severity of liver fibrosis and revealed a significant correlation with the stage of liver fibrosis [17]. Combining previous findings with our study, SIR_HBP_ and CEI_HBP_ after Gd-EOB-DTPA-enhanced MRI could be useful as novel imaging markers for estimating liver function.

SWE is performed using an ultrasound transducer, which emits high-frequency sound waves into the body to evaluate tissue elasticity, as stiffer tissues exhibit less deformation compared to softer tissues [18]. As a safe, inexpensive, and reproducible technique, SWE is used to evaluate the elasticity of liver tissue in various liver diseases [19]. Our results showed that patients with higher Child-Pugh levels showed elevated SWE levels. For diagnostic accuracy of Child-Pugh B and C and Child-Pugh C alone, the AUCs of SWE were 0.919 and 0.889, respectively, with both the sensitivity and specificity of SWE being higher than those of SIR_HBP_ and CEI_HBP_. Literature has also reported that liver stiffness measurement is independently associated with poor clinical outcomes [20] and improves the prognostic ability of biochemical response criteria, fibrosis scores, and prognostic models.

One limitation of SWE is that its results are influenced by the operator’s skill, the patient’s body constitution, and their compliance, which can reduce its reproducibility. SIR_HBP_ and CEI_HBP_ of MRI are independent of the operator’s skill. We suggest that, compared with SWE, Gd-EOB-DTPA-enhanced MRI can objectively reflect liver function in patients with PBC. We observed a significant difference between SIR_HBP_ and CEI_HBP_ values across different SWE groups; meanwhile, SWE was negatively correlated with SIR_HBP and_ CEI_HBP_. This indicates that Gd-EOB-DTPA-enhanced MRI and SWE have good consistency and can be used together to evaluate liver function. SWE can be used as a complementary method to Gd-EOB-DTPA-enhanced MRI.

APRI and FIB-4 are widely used for non-invasive fibrosis prediction in various liver diseases [21-23]. Wang *et al.* reported that the AUCs of APRI and FIB-4 were 0.816 and 0.83, respectively, for Scheuer stage ≥III [24]. SWE displayed a significant correlation with FIB-4 and APRI [25, 26]. SWE and Gd-EOB-DTPA-enhanced MRI demonstrated a remarkable diagnostic performance compared to FIB-4 and APRI in detecting liver fibrosis [27]. Another study reported that GPR was more sensitive than APRI and FIB-4 in detecting advanced fibrosis in patients with PBC [28]. Our results differ somewhat. GPR showed no significant differences across different Child-Pugh or SWE grades, and its differences between different CEI_HBP_ levels were not as significant as those of APRI and FIB-4, both the sensitivity and specificity of GPR being lower than those of APRI and FIB-4. According to our results, FIB-4 showed better consistency with liver function in non-invasive fibrosis models of PBC patients. We believe this may be due to GGT, a specific indicator reflecting bile duct injury, being more affected by liver inflammation activity in PBC patients. Since GPR is calculated based on GGT, its accuracy in evaluating liver fibrosis in PBC patients is not as good as APRI and FIB-4. Our results also showed that, for diagnostic accuracy of Child-Pugh B and C or Child-Pugh C, both sensitivity and specificity of Gd-EOB-DTPA-enhanced MRI and SWE were better than non-invasive fibrosis models.

Several limitations should be acknowledged. First, the sample size was relatively small; however, the proportions of patients based on Child-Pugh score showed similar distributions for the general population. Moreover, since histological correlation was not performed, larger and more diverse studies are necessary to validate these non-invasive examinations alongside their histological correlations. Furthermore, there were notably fewer patients in Child-Pugh B and C groups compared to Child-Pugh A, potentially reducing statistical power for advanced disease assessment.

## CONCLUSION

This study confirmed that Gd-EOB-DTPA-enhanced MRI is an effective and objective method for evaluating liver function in patients with PBC. SIR_HBP_ and CEI_HBP_ could be useful as novel imaging biomarkers for assessing liver function. Both MRI and SWE outperformed the serum-based models in terms of sensitivity and specificity, reinforcing the potential of imaging in clinical decision-making.

## Figures and Tables

**Fig. (1):**
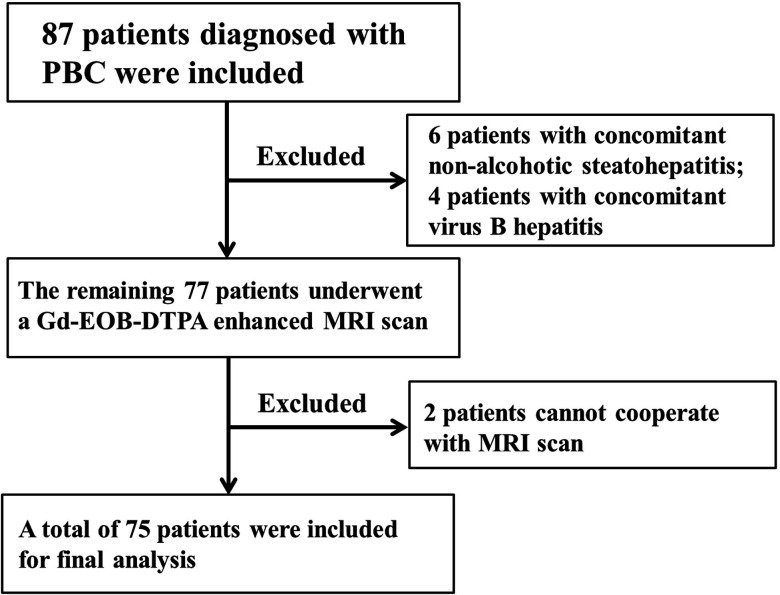
Flowchart of the study population.

**Fig. (2):**
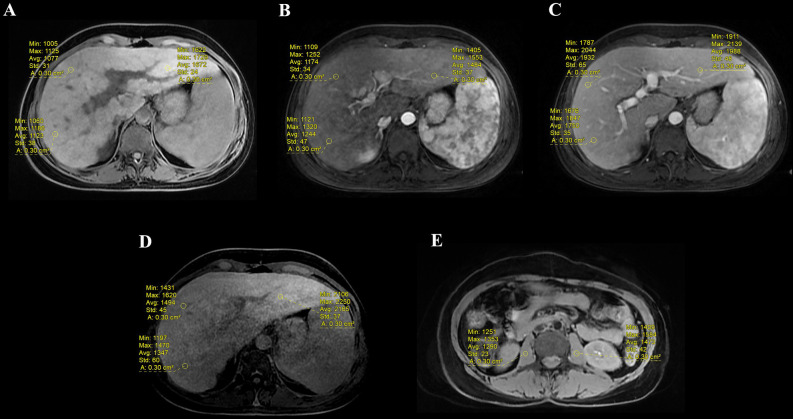
Signal intensity (SI) values in T1-weighted images before and after the injection of Gd-EOB-DTPA with operator-defined region-of-interest (ROI) drawn on the liver lobe. (**A**) SI on precontrast; (**B**) SI on portal venous phase (PVP); (**C**) SI on balance phase (EP); (**D**) SI on balance phase (HBP); (**E**) SI on paravertebral muscle (PM).

**Table 1:** Clinical characteristics of the participants.

Age (years) ^a^								56(47, 63.5)		
Male (*n*, %)								13(17.3%)		
Clinical data			WBC (×10^9^/L) ^a^					4.38(3.30, 6.10)		
			PLT (×10^9^/L) ^a^					136(69, 225)		
			ALT (U/L) ^a^					40(30, 102.5)		
			AST (U/L) ^a^					49(27, 74)		
			GGT (U/L) ^a^					154(61, 348)		
			AKP (U/L) ^a^					178(125, 375)		
MRI data			SIR_pvp_^a^					2.46(2.07, 3.19)		
			SIR_EP_^a^					2.77(2.25, 3.36)		
			SIR_HBP_^a^					2.82(2.09, 4.05)		
			CEI_PVP_^a^					0.65(0.41, 0.97)		
			CEI_EP_^a^					0.74(0.50, 1.07)		
			CEI_HBP_^a^					0.75(0.49, 1.32)		
SWE ^a^								10.6(6.1, 15.65)		
Non-invasive fibrosis models			APRI ^a^					1.2(0.67, 2.23)		
			FIB-4 ^a^					2.53(1.44, 7.09)		
			GPR ^a^					4.23(1.85, 7.83)		
Child-Pugh (*n*, %)			A					37(49.3%)		
			B					22(29.3%)		
			C					16 (21.3%)		

**Abbreviations:**WBC: white blood cell, PLT: platelets count, ALT: alanine aminotransferase, AST: aspartate aminotransferase, ALP: alkaline phosphatase, GGT: Gamma-glutamyl transpeptidase, SIR: signal intensity ratio, CEI: contrast enhancement index, PVP: portal venous phase, EP: equilibrium phase, HBP: hepatobiliary phase, SWE: real-time shear wave elastography, APRI: aspartate transaminase to platelet ratio index, FIB-4: fibrosis index based on the 4 factors, GPR: gamma-glutamyl transferase to platelet ratio. ^a^ data were described as median (P_25_, P_75_).

**Table 2:** Comparison of Gd-EOB-DTPA-enhanced MRI, SWE, and non-invasive fibrosis models across different Child-Pugh grades.

		Child-Pugh			N	Median (P_25_, P_75_)				H			*p*	
Age (years)		A			37	53.0(47.0, 57.0)				12.735			0.002*	
		B			22	52.5(46.0, 58.0)								
		C			16	69.5(56.0, 76.0)								
SIR_pvp_		A			37	2.86(2.09, 3.32)				4.821			0.090	
		B			22	2.13(2.41, 3.27)								
		C			16	2.32(1.95, 2.57)								
SIR_EP_		A			37	3.25(2.30, 3.60)				5.443			0.066	
		B			22	2.56(2.02, 3.15)								
		C			16	2.50(2.25, 3.01)								
SIR_HBP_		A			37	2.88(3.07, 4.76)				21.473			<0.001**	
		B			22	2.29(2.00, 3.32)								
		C			16	1.92(2.15, 2.57)								
CEI_PVP_		A			37	0.77(0.45, 1.01)				4.160			0.125	
		B			22	0.71(0.35, 1.02)								
		C			16	0.47(0.31, 0.69)								
CEI_EP_		A			37	0.84(0.63, 1.21)				5.190			0.078	
		B			22	0.58(0.50, 0.85)								
		C			16	0.72(0.49, 0.89)								
CEI_HBP_		A			37	1.33(0.90, 1.83)				25.712			<0.001**	
		B			22	0.61(0.35, 0.84)								
		C			16	0.50(0.40, 0.55)								
SWE		A			37	7.20(5.38, 9.10)				42.216			<0.001**	
		B			22	11.75(10.20, 14.73)								
		C			16	19.10(14.68, 20.93)								
APRI		A			37	0.86(0.54, 1.45)				12.415			0.002*	
		B			22	1.52(0.60, 2.78)								
		C			16	2.17(1.09, 4.28)								
FIB-4		A			37	1.76(1.21, 3.24)				24.286			<0.001**	
		B			22	2.51(1.58, 5.70)								
		C			16	10.21(4.93, 17.85)								
GPR		A			37	2.84(1.69, 5.42)				3.664			0.160	
		B			22	7.93(1.45, 13.16)								
		C			16	4.44(1.78, 6.83)								

**Abbreviations:**SIR: signal intensity ratio, CEI: contrast enhancement index, PVP: portal venous phase, EP: equilibrium phase, HBP: hepatobiliary phase, SWE: real-time shear wave elastography, APRI: aspartate transaminase to platelet ratio index, FIB-4: fibrosis index based on the 4 factors, GPR: gamma-glutamyl transferase to platelet ratio. *p<0.01, **p<0.001.

**Table 3:** Comparison of Gd-EOB-DTPA-enhanced MRI and non-invasive fibrosis models across different SWE grades.

	SWE grades			N			Median (P_25_, P_75_)		H			p	
Age (years)	SWE≤7.0			20			50.0(47.0, 57.0)		5.265			0.072	
	7.0<SWE≤14			35			56.0(47.0, 60.0)						
	SWE>14			20			57.5(52, 75.25)						
SIR_pvp_	SWE≤7.0			20			2.86(2.25, 3.41)		3.799			0.150	
	7.0<SWE≤14			35			2.76(2.08, 3.23)						
	SWE>14			20			2.21(1.95, 2.68)						
SIR_EP_	SWE≤7.0			20			3.36(2.29, 3.70)		3.918			0.141	
	7.0<SWE≤14			35			2.81(2.23, 3.30)						
	SWE>14			20			2.56(2.25, 3.08)						
SIR_HBP_	SWE≤7.0			20			3.93(2.41, 4.86)		10.531			0.005	
	7.0<SWE≤14			35			2.98(2.20, 3.83)						
	SWE>14			20			2.15(1.83, 2.67)						
CEI_PVP_	SWE≤7.0			20			0.74(0.44, 0.99)		2.509			0.357	
	7.0<SWE≤14			35			0.74(0.41, 1.00)						
	SWE>14			20			0.57(0.30, 0.76)						
CEI_EP_	SWE≤7.0			20			0.78(0.53, 1.22)		1.392			0.498	
	7.0<SWE≤14			35			0.75(0.51, 1.07)						
	SWE>14			20			0.67(0.50, 0.85)						
CEI_HBP_	SWE≤7.0			20			1.12(0.54, 1.69)		9.240			0.010	
	7.0<SWE≤14			35			0.94(0.50, 1.36)						
	SWE>14			20			0.53(0.40, 0.70)						
APRI	SWE≤7.0			20			1.04(0.43, 1.65)		5.466			0.065	
	7.0<SWE≤14			35			1.00(0.63, 1.62)						
	SWE>14			20			1.76(0.91, 3.85)						
FIB-4	SWE≤7.0			20			2.21(0.86, 3.26)		14.097			0.001	
	7.0<SWE≤14			35			1.89(1.37, 4.35)						
	SWE>14			20			7.51(2.71, 12.50)						
GPR	SWE≤7.0			20			4.30(1.84, 6.22)		0.011			0.994	
	7.0<SWE≤14			35			3.31(1.64, 8.85)						
	SWE>14			20			4.53(1.5, 7.53)						

**Abbreviations:** SIR: signal intensity ratio, CEI: contrast enhancement index, PVP: portal venous phase, EP: equilibrium phase, HBP: hepatobiliary phase, SWE: real-time shear wave elastography, APRI: aspartate transaminase to platelet ratio index, FIB-4: fibrosis index based on the 4 factors, GPR: gamma-glutamyl transferase to platelet ratio.

## Data Availability

The data of current study are available from corresponding authors, [Z.W] and [Z.Y], on a reasonable request.

## LIST OF ABBREVIATIONS

Gd-EOB-DTPA: Gadolinium ethoxybenzyl diethylenetriamine pentaacetic acid
MRI: Magnetic resonance imaging
PBC: Primary biliary cholangitis
SWE: Shear wave elastography
SIR: Signal intensity ratio
CEI: Contrast enhancement index
PVP: Portal venous phase
EP: Equilibrium phase
HBP: Hepatobiliary phase
AUC: Area under the curve
AST: Aspartate transaminase
ALT: Alanine transaminase
ALP: Alkaline phosphatase
GGT: Gamma-glutamyl transferase
PLT: Platelets

## STANDARDS OF REPORTING

STROBE guidelines were followed.
